# Surrogate resilience and clinical titration of presence in the open intensive care unit: a systematic narrative synthesis

**DOI:** 10.1016/j.ijnsa.2026.100583

**Published:** 2026-06-08

**Authors:** Ali Bahramifar, Amir Vahedian-Azimi

**Affiliations:** a Trauma Research Center, Medicine Faculty, Baqiyatallah University of Medical Sciences, Tehran, Iran; b Nursing Care Research Center, Clinical Sciences Institute, Nursing Faculty, Baqiyatallah University of Medical Sciences, Tehran, Iran

**Keywords:** Intensive care units, Family-centered care, Surrogate decision-making, Post-intensive care syndrome-family, Psychological resilience

## Abstract

**Background:**

Open-access intensive care unit policies prioritize family integration. However, these guidelines may not fully account for the physical and psychological demands placed on surrogate decision-makers. Continuous, unregulated family presence is associated with physical exhaustion and cognitive fatigue. This burden may negatively affect the decisional capacity these policies intend to support.

**Objectives:**

This study aimed to evaluate the evidence regarding the psychological burden on intensive care unit surrogates and to construct a resilience framework based on the Conservation of Resources theory.

**Design and methods:**

This systematic narrative synthesis (Resilience Ecosystem for Surrogate Titration, Overload Recovery, and Engagement) combined the methodological rigor of systematic identification, utilizing structured database searches, dual screening, and standardized quality appraisal, with narrative thematic analysis. A multi-database search (PubMed, Scopus, Web of Science) identified relevant literature published through February 2026. After quality appraisal, data from 35 articles were analyzed using thematic synthesis and mapped onto the Conservation of Resources framework.

**Results:**

Findings suggest that current evaluation metrics often equate visitation duration with quality of care, which may not account for the physiological limits of surrogates. The synthesis identifies "Compulsive Hyper-engagement," a state of cognitive fatigue associated with continuous, unstructured visitation. To address this, the framework proposes the Clinical Titration of Family Presence, which uses structured rest periods ("Restorative Dosing") to regulate the intensity of family engagement. Furthermore, a Pan-Dimensional Matrix for Surrogate Resilience is introduced. This matrix categorizes protective interventions across five dimensions: physiological infrastructure, cognitive support, psycho-spiritual frameworks, social architecture, and technological connectivity.

**Conclusions:**

The synthesized evidence identifies a plausible risk that unrestricted intensive care unit visitation without structural support may contribute to surrogate exhaustion. To preserve decisional capacity, clinical practice may benefit from shifting toward actively structuring family resilience. Implementing the Clinical Titration of Presence suggests providing scheduled rest intervals. This approach aims to reduce surrogate guilt and support their role as capable partners in shared decision-making.

**Registration:**

Not applicable. (As a narrative synthesis, prospective registration is not required; however, an internal methodological protocol was followed).

## Graphical abstract

**Figure:**
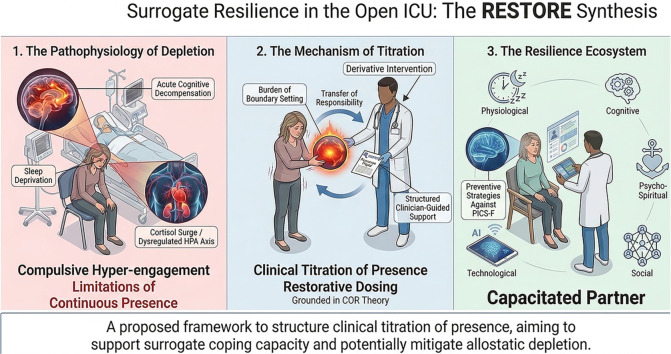

**Synthesis: A Conceptual Framework for Surrogate Resilience in the Open Intensive Care Unit**

This three-panel visualization illustrates the conceptual transition from surrogate physiological depletion to a framework of sustainable engagement.

**Panel 1** (**The Pathophysiology of Depletion**) outlines the potential neurocognitive and metabolic correlates of continuous, unstructured Intensive Care Unit presence. It addresses the *Metric Fallacy,* indicating how sustained allostatic load, associated with Hypothalamic-Pituitary-Adrenal (HPA) axis activation and sleep deprivation, may contribute to *Compulsive Hyper-engagement* and acute cognitive alterations.

**Panel 2** (**The Mechanism of Titration**) depicts the operationalization of the *Clinical Titration of Presence*. Grounded in the Conservation of Resources (COR) theory, this phase illustrates a structured clinical approach to facilitate a Transfer of Responsibility. By conceptualizing respite as *Restorative Dosing* (Rx REST), the intervention aims to assist the surrogate with the Burden of Boundary-Setting.

**Panel 3** (**The Resilience Ecosystem**) outlines the objective of this framework: supporting the surrogate as a *Capacitated Partner*. This state is supported by a five-dimensional structural matrix (Physiological, Cognitive, Psycho-Spiritual, Social, and Technological), designed to help preserve cognitive capacity and potentially mitigate the risk of Post-Intensive Care Syndrome-Family (PICS-F).

**What is already known**

• Open-access Intensive Care Unit policies facilitate family integration but often introduce unrecognized physiological and psychological demands on surrogate decision-makers.
• Unmitigated allostatic load and severe sleep deprivation during prolonged bedside vigils are strongly associated with compromised executive function and Post-Intensive Care Syndrome-Family.

Alt-text: Unlabelled box dummy alt text

**What this paper adds**

• Introduces the concept of Compulsive Hyper-engagement to define the acute cognitive decompensation surrogates experience during unstructured, continuous intensive care unit visitation.
• Proposes the Clinical Titration of Presence as an active resilience engineering strategy, utilizing restorative dosing to ethically unburden surrogates and preserve their decisional capacity.
• Presents a Pan-Dimensional Matrix for Surrogate Resilience, systematically mapping evidence-based interventions across five ecosystemic domains to transition surrogates into capacitated partners.

Alt-text: Unlabelled box dummy alt text

## 1 Introduction

Over the past two decades, critical care has increasingly transitioned toward open-access intensive care unit policies (Berwick and Kotagal, 2004). While reducing visitation restrictions may facilitate family integration (Davidson et al., 2017), this approach can also introduce significant physical and mental demands on patient surrogates. While 'surrogate' specifically refers to the individual(s) holding legal or functional decisional authority (Hwang et al., 2025), the psychological and physiological effects described in this framework apply broadly to family members experiencing sustained bedside presence. The widespread adoption of unrestricted access policies has sometimes outpaced the evaluation of the psychological effects associated with prolonged surrogate presence (Day et al., 2013). Current hospital frameworks often equate the duration of physical presence with optimal family engagement. This emphasis may inadvertently contribute to surrogate exhaustion and physiological depletion (Needham et al., 2012).

Empirical literature regarding intensive care unit families consistently reports psychological distress, sleep disruption, and decisional burden (Day et al., 2013; Beesley et al., 2018). While these states are often associated with hypothalamo-pituitary-adrenocortical (HPA) axis activation and increased allostatic load in general stress models, specific metabolic evidence in the intensive care unit surrogate population is frequently inferred from broader physiological frameworks (Arnsten, 2009).

While contemporary standards, such as the 2024 Society of Critical Care Medicine (SCCM) guidelines, advocate for flexible visitation and encompass holistic support, including mental health, spiritual care, and clinician training, their operationalization remains a challenge (Davidson et al., 2017). Existing frameworks provide limited concrete guidance for determining the specific threshold at which sustained family presence may become burdensome for the surrogate decision-maker. This lack of operational boundaries, coupled with the implicit expectation to remain available, may contribute to a state conceptualized herein as 'Compulsive Hyper-engagement'—a proposed theoretical construct describing a sustained hypervigilant presence and a perceived obligation to remain bedside, rather than a formal clinical diagnosis. This state is characterized by a surrogate’s sustained vigilance and difficulty disengaging from the clinical environment. For example, a surrogate may remain at the bedside for consecutive days with minimal sleep, driven by an obligation to monitor clinical events.

Beyond normative situational distress, this state may be associated with cognitive alterations. Sustained stress responses, combined with sleep deprivation, may increase allostatic load and negatively affect executive function (Beesley et al., 2018; Arnsten, 2009). These alterations may manifest as impaired attention, working memory, and emotional regulation (Rahimibashar et al., 2022). Such cognitive changes present significant clinical challenges, as surrogates are frequently required to participate in complex decision-making; compromised executive control and decisional fatigue may increase the risk of decisional delay or impulsivity (Pignatiello et al., 2020). Furthermore, prolonged exposure under these conditions has been associated with Post-Intensive Care Syndrome-Family (PICS-F), potentially contributing to subsequent anxiety and Post-Traumatic Stress Disorder (PTSD) (Blot et al., 2025; Davidson et al., 2012).

The underrecognition of these effects may relate to current evaluation metrics. The efficacy of family integration is frequently assessed via access metrics (e.g., visitation hours) and satisfaction surveys. These tools may lack the sensitivity required to detect surrogate exhaustion (Fumis et al., 2015; Heyland et al., 2002). These limitations suggest a need to reevaluate the structure of family participation programs to minimize unintended psychological morbidity (Rahimi-Bashar et al., 2024). Surrogates may report high satisfaction based on uninterrupted access, potentially equating prolonged physical presence with optimal patient advocacy (Fumis et al., 2015). In the absence of formalized protocols addressing the perceived obligation to remain during clinical interventions (Jabre et al., 2013), institutional practices may inadvertently contribute to surrogate burnout. Without monitoring surrogate physical reserve (White et al., 2018), the responsibility for boundary-setting may fall upon individuals already experiencing significant psychological stress (Cameron et al., 2016).

Transitioning toward sustainable family engagement requires conceptualizing the Intensive Care Unit as a supportive environment for both surrogates and patients. Despite emerging discussions regarding the regulation of family presence, there remains a need for evidence-based frameworks designed to support resilience in ICU families. Therefore, this narrative review aims to synthesize current literature regarding surrogate psychological burden and potential restorative interventions. Utilizing Stevan E. Hobfoll’s Conservation of Resources (COR) theory (Hobfoll, 1989) as a theoretical framework, this review maps empirical evidence onto a proposed Pan-Dimensional Matrix for Surrogate Resilience. This matrix integrates physiological, cognitive, psycho-spiritual, social, and technological domains. The RESTORE (Resilience Ecosystem for Surrogate Titration, Overload Recovery, and Engagement) framework aims to assist clinicians in supporting the physical and psychological capacity of surrogate decision-makers.

## 2 Methods

### 2.1 Study design and methodological framework

To address the selection biases sometimes associated with conventional narrative reviews, the Resilience Ecosystem for Surrogate Titration, Overload Recovery, and Engagement (RESTORE) framework was designed as a systematic narrative synthesis. This approach integrates the rigor of systematic identification, including structured search protocols, dual screening, and quality appraisal, with the conceptual breadth of narrative thematic analysis. The methodology utilized the Scale for the Assessment of Narrative Review Articles (SANRA) specifically as a quality assessment tool for the narrative components (**Electronic Supplementary Material S1**) (Baethge et al., 2019). Furthermore, to support reproducibility, the search and screening protocols were informed by the Joanna Briggs Institute (JBI) methodology (Peters et al., 2020).

Although prospective databases (e.g., International Prospective Register of Systematic Reviews (PROSPERO)) do not register narrative and scoping reviews, an *a priori* methodological protocol was internally formalized and followed throughout the study to mitigate *post hoc* reporting biases. This methodological approach facilitates the thematic exploration characteristic of a narrative review while incorporating the structured parameters of a systematic search. Stevan E. Hobfoll’s Conservation of Resources (COR) theory (Hobfoll, 1989) was utilized as the theoretical framework to guide the evaluation and conceptual mapping of the literature.

### 2.2 Search strategy and information sources

A structured literature search was conducted across three academic databases: PubMed (National Library of Medicine), Scopus (Elsevier), and Web of Science (Core Collection, Clarivate Analytics). These databases were selected to provide multidisciplinary coverage across medicine, nursing, and the psychosocial sciences, aiming to capture the necessary conceptual breadth (Bramer et al., 2017). The search spanned from the inception of each database to February 22, 2026, with the final automated query executed on February 24, 2026. To support literature saturation, database queries were supplemented by forward and backward citation tracking of all included manuscripts.

The search syntax was developed in consultation with an academic medical research librarian, integrating standardized controlled vocabularies (e.g., Medical Subject Headings (MeSH) terms) with relevant free-text keywords. To capture established literature and emerging concepts, the search matrix was structured around four domains: (1) Setting/Context (spanning terms like "Intensive Care Units" to "Green Intensive Care Unit"); (2) Population/Actors (ranging from "Family" and "Caregivers" to "Capacitated Partners"); (3) Phenomena of Interest (contrasting conventional descriptors like "Vigilance" with "Compulsive Hyper-engagement" and "Clinical Titration of Presence"); and (4) Outcomes (extending beyond classical endpoints such as " Post-Intensive Care Syndrome-Family (PICS-F)" to encompass proactive interventions like active resilience engineering). Boolean operators (AND, OR) were applied to intersect these domains. To maintain data integrity during the export process, database metadata were extracted, aggregated, and formatted into comma-separated values (CSV) files (McKinney, 2010) (the technical specifics of the automated extraction scripts are detailed in **Electronic Supplementary Material S2**).

### 2.3 Eligibility criteria

To standardize the selection process, specific inclusion and exclusion criteria were established *a priori*.

**Inclusion Criteria:** (1) Peer-reviewed primary research (randomized controlled trials, observational cohorts, cross-sectional studies), qualitative investigations, theoretical frameworks, clinical practice guidelines, conceptual reviews, and peer-reviewed commentaries; (2) Studies explicitly examining the psychological burden, engagement patterns (including hyper-vigilance), or resilience interventions among surrogate decision-makers/family members. Literature assessing family members in general was included provided that the reported outcomes (e.g., allostatic load, sleep disruption) directly intersected with the cognitive and physiological capacity required for surrogate decision-making and patient advocacy; (3) Contextualized exclusively within adult Intensive Care Units; and (4) Published in the English language (Morrison et al., 2012).

**Exclusion Criteria:** (1) Studies strictly confined to pediatric or neonatal intensive care settings; (2) Grey literature, unindexed preprints, non-peer-reviewed editorials (distinct from peer-reviewed commentaries published in indexed journals), and conference abstracts lacking full-text data; and (3) Studies focusing solely on patient-centric outcomes without explicitly evaluating the psychological or systemic impact on the family unit.

### 2.4 Study selection and procedural flow

In lieu of a visual Preferred Reporting Items for Systematic Reviews and Meta-Analyses (PRISMA) 2020 flow diagram (Page et al., 2021), the stepwise progression of record identification and screening is detailed below. The initial search yielded 2267 records (PubMed: n = 1108; Web of Science: n = 797; Scopus: n = 362). Following data importation, 645 duplicate records were identified and removed using EndNote™ v21 (Clarivate Analytics), supplemented by manual cross-verification.

The screening process was conducted in two phases. Prior to formal screening, a pilot exercise was performed on a random subsample of 10 abstracts to calibrate the application of the eligibility criteria and ensure consistent interpretation between the reviewers. Initially, the primary investigator (AVA) and an independent methodology consultant screened the titles and abstracts of the 1622 deduplicated records against the predefined eligibility criteria. Consequently, 1430 records were excluded, leaving 192 reports sought for retrieval. Twelve reports were unretrievable, resulting in 180 articles subjected to full-text appraisal. Following this evaluation, 145 articles were excluded based on *a priori* parameters (n = 76 for an incompatible phenomenon of interest; n = 43 for ineligible population demographics; n = 26 for an incongruent clinical setting). Subsequent forward and backward citation tracking (Greenhalgh and Peacock, 2005) of the eligible full-texts yielded no additional articles meeting the inclusion criteria. Ultimately, a final cohort of 35 articles was deemed eligible for quality appraisal, data extraction, and synthesis. Discrepancies between the reviewers were resolved through consensus or via adjudication by an independent senior methodologist.

### 2.5 Quality appraisal and risk of bias assessment

Quality appraisal was conducted independently by the primary investigator and the methodology consultant utilizing design-specific Joanna Briggs Institute (JBI) Critical Appraisal Checklists (e.g., specific tools for randomized controlled trials, cross-sectional, and qualitative studies) (Peters et al., 2020). Discrepancies in assigned ratings were resolved through consensus or arbitration by the independent senior methodologist. Consistent with the exploratory nature of a systematic narrative synthesis, studies were not excluded based solely on lower quality scores. Instead, the appraisal outcomes directly influenced conceptual weighting; studies exhibiting significant methodological limitations (e.g., high risk of bias or small sample sizes) were assigned lower interpretive weight. Findings from these specific studies were explicitly contextualized as hypothesis-generating, rather than confirmatory, during the thematic synthesis and subsequent discussion.

### 2.6 Data extraction and synthesis strategy

Data synthesis was conducted using a hybrid inductive-deductive thematic approach to mitigate the risk of circular reasoning (Fereday and Muir-Cochrane, 2006). Thematic coding and cross-tabulation were managed using Microsoft Excel and NVivo™ qualitative data analysis software. Initially, empirical findings were extracted and coded inductively from the 35 included articles to identify recurring patterns without a pre-existing theoretical template. Subsequently, these emergent themes were deductively mapped onto the Conservation of Resources (COR) theory (Hobfoll, 1989), utilizing core concepts such as 'Resource Loss Spirals' and 'Resource Investment.' This two-stage process ensured that the RESTORE framework remained grounded in primary data while benefiting from the structural rigor of Conservation of Resources (COR) theory (**Figure S1**). Disagreements regarding code assignment were resolved through a three-tier adjudication process involving a senior independent methodologist. Furthermore, disconfirming evidence, including studies identifying vigilance as a positive coping mechanism, was systematically addressed by incorporating these findings into the 'Paradox of Agency' and cultural context sections to establish the framework's boundary conditions. While Table 1 utilizes the resulting Pan-Dimensional Matrix labels for clinical clarity, which encompasses the Physiological, Cognitive, Psycho-Spiritual, Social, and Technological domains, the underlying mapping reflects a genuine application of COR principles, specifically identifying areas of resource depletion and restorative gain. This matrix structures the evidence base into five interconnected dimensions: (1) Physiological & Environmental Infrastructure; (2) Cognitive & Information Prosthetics; (3) Psycho-Spiritual Scaffolding; (4) Social & Logistical Architecture; and (5) Technological & Predictive Connectivity. This synthesis facilitated the translation of empirical findings into a framework intended to support active resilience engineering within the intensive care setting.

**Table 1:** Characteristics of the 35 included studies and conceptual mapping to the conservation of resources (COR) framework.

Authorship (Year) [Ref]	Geographical context	Design & source type	Primary conceptualization of surrogate engagement	Conservation of resources (COR) theoretical framework mapping
(Agård and Lomborg, 2011)	Europe	Qualitative Ethnography	Nurses' Decision-making & Flexible Visitation	Baseline Deficit (Metric Fallacy)
(Schmidt and Azoulay, 2013)	Europe	Observational Commentary	Surrogate Sleep Deprivation & Physiological Burden	Physiological & Environmental Infrastructure
(Wilson et al., 2019)	North America	Conceptual Review	ICU Humanization & Ethical Unburdening	Cognitive & Psycho-Spiritual Scaffolding
(Eftekhari et al., 2025)	Middle East / Global	Narrative Synthesis	AI-Driven Communication & Empathic Listening	Technological & Predictive Connectivity
(Rahimi-Bashar et al., 2022)	Middle East	Multicenter RCT	Long-term Life Satisfaction & Rehabilitation	Social & Logistical Architecture
(Xyrichis et al., 2021)	Europe	Systematic Review	Family Involvement Interventions in Critical Care	Cognitive & Information Prosthetics
(Jafarabadi et al., 2020)	Middle East	Systematic Review & Meta-analysis	Stress Reduction via Progressive Muscle Relaxation	Physiological & Environmental Infrastructure
(Vahedian-Azimi et al., 2014)	Middle East	Randomized Controlled Trial (RCT)	Non-pharmacological Somatic Interventions (Massage)	Physiological & Environmental Infrastructure
(Ayenew et al., 2025)	Africa / Global	Systematic Review & Meta-analysis	Prevalence of PICS-F & ICU-survivor Sequelae	Baseline Deficit (Allostatic Load)
(Checa-Checa et al., 2025)	Europe / South America	Systematic Review	Family Support Strategies & Structural Needs	Social & Logistical Architecture
(Lucchini et al., 2025)	Global	Conceptual Framework	Personalized ICU Care Evolution	Pan-Dimensional Matrix (Baseline)
(Shirasaki et al., 2024)	Asia	Comprehensive Review	PICS-F Etiology & Surrogate Depletion	Psycho-Spiritual Scaffolding
(Ashghab et al., 2024)	Middle East	Systematic Review & Meta-analysis	Sleep Quality Improvement Interventions	Physiological & Environmental Infrastructure
(Hamilton et al., 2025)	North America	Clinical Guidelines (SCCM)	Adult ICU Architectural Design Standards	Physiological & Environmental Infrastructure
(Bhatt et al., 2025)	Global	Scoping Review	Cultural & Socioeconomic Determinants of Satisfaction	Cognitive Prosthetics (Metric Fallacy)
(Hwang et al., 2025)	North America	Clinical Guidelines (SCCM)	Family-Centered Care Protocols	Cognitive & Information Prosthetics
(Lautrette et al., 2007)	Europe	Randomized Controlled Trial (RCT)	Structured Communication Strategy (VALUE Protocol)	Cognitive & Information Prosthetics
(Vahedian-Azimi et al., 2016)	Middle East	Randomized Controlled Trial (RCT)	Family-Centered Empowerment Model Efficacy	Cognitive & Information Prosthetics
(Boehm et al., 2020)	North America	Implementation Study	ICU Peer Support Programs	Psycho-Spiritual Scaffolding
(Stone, 2018)	North America	Intervention Study	Code Lavender & Rapid Staff Support	Psycho-Spiritual Scaffolding
(Bashar et al., 2018)	Middle East	Cross-Sectional Study	Spiritual Health Diagnostics in Critical Care	Psycho-Spiritual Scaffolding
(Kang et al., 2019)	Asia	Meta-analysis	Meaning-Centered Existential Interventions	Psycho-Spiritual Scaffolding
(Vahedian-Azimi et al., 2023)	Middle East	Cross-Sectional Study	Spirituality as a Buffer for ICU Trauma Memories	Psycho-Spiritual Scaffolding
(Farzanegan et al., 2021)	Middle East	Prospective Observational	Religiosity Impact on Delirium Severity	Psycho-Spiritual Scaffolding
(Khandelwal et al., 2025)	North America	Observational Cohort	Financial Toxicity & Secondary Hardship	Social & Logistical Architecture
(Pell et al., 2015)	North America	Observational Study	EHR Access Transparency during Hospitalization	Technological & Predictive Connectivity
(Rosenthal et al., 2024)	North America	Mixed Methods	Inpatient Telehealth for Remote Engagement	Technological & Predictive Connectivity
(Arabfard et al., 2025)	Middle East / Global	Theoretical Framework	AI-Integrated Green ICU Models	Technological & Predictive Connectivity
(Pandian et al., 2026)	Global	Systematic Review & Synthesis	AI-Driven Communication in Delirium Care	Technological & Predictive Connectivity
(Alhani et al., 2022)	Middle East	Systematic Review & Meta-analysis	Family-Centered Empowerment & Quality of Life	Social & Logistical Architecture
(Cai et al., 2026)	Asia	Umbrella Review	Non-Pharmacological PICS Interventions	Physiological & Cognitive Prosthetics
(Vahedian-Azimi, 2025)	Middle East	Review / Commentary	Cultural Competence & Family Conflict Resolution	Social & Logistical Architecture
(Giannini et al., 2013)	Europe	Before-and-After Study	Impact of Liberalized Visitation on ICU Staff	Baseline Context (Clinician Distress)
(Khaghanizadeh et al., 2023)	Middle East	Randomized Controlled Trial (RCT)	Ethical Decision-Making Training vs. Moral Distress	Psycho-Spiritual & Cognitive Scaffolding
(Vahedian-Azimi et al., 2019)	Middle East	Cross-Sectional Study	Effects of Bedside Stress on Critical Care Nurses	Baseline Context (Clinician Distress)

a. Population: All studies involve adult ICU family members/surrogates unless otherwise specified.; b. Mapping: All COR/RESTORE mappings were assigned by the authors during the thematic synthesis. **Abbreviations: AI:** Artificial Intelligence; **COR:** Conservation of Resources; **EHR:** Electronic Health Record; **ICU:** Intensive Care Unit; **PICS:** Post-Intensive Care Syndrome; **PICS-F:** Post-Intensive Care Syndrome-Family; **RCT:** Randomized Controlled Trial; **SCCM:** Society of Critical Care Medicine; **VALUE:** Value family statements, Acknowledge emotions, Listen, Understand the patient as a person, Elicit family questions (Structured communication protocol).

## 3 Results

### 3.1 Literature retrieval and study characteristics

As detailed in the methodological protocol, the search yielded an initial corpus of 2267 records. Following deduplication and a two-phase screening process, a final cohort of 35 manuscripts met the eligibility criteria for quality appraisal and data extraction. To support methodological transparency and align with the Resilience Ecosystem for Surrogate Titration, Overload Recovery, and Engagement (RESTORE) narrative synthesis reporting standards, the characteristics, methodological designs, and conceptual mappings of these 35 studies to the Conservation of Resources (COR) theoretical framework are detailed in Table 1. As specified in Table 1, the evidence base encompasses diverse source types and populations, with each study subjected to standardized quality appraisal. All conceptual mappings to the Resilience Ecosystem for Surrogate Titration, Overload Recovery, and Engagement (RESTORE) domains were assigned by the authors based on the iterative coding process detailed in the methodology.

The thematic synthesis of these manuscripts, analyzed using the Conservation of Resources (COR) theory, identifies a tension between the model of continuous physical presence and the concept of sustainable surrogate engagement. The following sections present the synthesized empirical evidence alongside our proposed theoretical interpretations and the resulting hypothesis-generating framework. The synthesized findings are categorized into the pathophysiology of surrogate fatigue, limitations of current evaluation metrics, the operationalization of clinical titration, and the proposal of a five-dimensional resilience framework.

### 3.2 Domain-by-domain synthesis: methodological pathway and coding translation

To elucidate the intermediate translational pathway from primary evidence to the Resilience Ecosystem for Surrogate Titration, Overload Recovery, and Engagement (RESTORE) framework, a domain-by-domain synthesis was conducted, linking extraction codes and appraisal judgments to the five domains. Methodological appraisal influenced coding weight; randomized controlled trials (RCTs) informed the *Physiological & Environmental Infrastructure* and *Cognitive Prosthetics* domains, validating interventions like chronobiological lighting and structured communication protocols. Conversely, qualitative investigations and ethnographies, appraised for phenomenological depth, primarily governed the *Psycho-Spiritual Scaffolding* and *Social Architecture* domains, capturing the subjective nuances of existential distress and financial toxicity.

During the inductive phase, 142 distinct operational codes were extracted from the 35 sources. Following adjudication, these were deductively consolidated into the five Conservation of Resources (COR) domains. For instance, empirical findings regarding 'information asymmetry' and 'prognostic confusion' were universally coded under 'Resource Loss' and mapped to the Cognitive domain, whereas interventions describing 'peer mentorship' were coded as 'Gain Caravans' within the social domain. Studies exhibiting methodological limitations (e.g., small cross-sectional cohorts) were not utilized to establish primary mechanisms but were mapped as supplementary corroboration within the Technological Connectivity domain. This appraisal-weighted translation ensures that each dimension of the framework is proportionally anchored to the most corresponding study designs (**Figure S2**).

### 3.3 The pathophysiology of compulsive hyper-engagement

The synthesized evidence suggests that while current guidelines (Davidson et al., 2017) emphasize unrestricted access within patient-centered care, they often lack physiological thresholds to identify when continuous familial presence may become unsustainable. To address this gap, this synthesis introduces 'Compulsive Hyper-engagement' as an author-proposed conceptual metaphor. This construct is defined herein as a sustained hypervigilant presence—a state in which a surrogate’s vigilance appears driven by a perceived moral obligation or psychological difficulty to disengage, rather than specific clinical necessity, often persisting despite emerging physical or cognitive exhaustion. This framework is intended to facilitate the clinical identification of these observed engagement patterns. While empirical studies confirm significant psychological burden and sleep deprivation in Intensive Care Unit surrogates, the proposed underlying mechanisms, such as metabolic failure or catecholamine variability, are largely extrapolated from general stress physiology (Beesley et al., 2018; Arnsten, 2009). These hypothesized effects are considered plausible factors that may contribute to the observed allostatic load and subsequent decline in executive function (Rahimibashar et al., 2022). Recognizing these cognitive changes is relevant because surrogates frequently participate in complex decision-making (e.g., processing probabilistic data, discussing life-sustaining therapies). In a state of allostatic overload, their functional capacity may be compromised, potentially increasing susceptibility to impulsivity or decisional delay (Pignatiello et al., 2020). Furthermore, prolonged Intensive Care Unit exposure under these conditions has been identified as a potential contributor to Post-Intensive Care Syndrome-Family (PICS-F), which is associated with long-term anxiety and Post-Traumatic Stress Disorder (PTSD) (Blot et al., 2025; Davidson et al., 2012). Within the proposed framework, facilitating continuous presence without providing structural support for rest is viewed as a potential contributor to surrogate exhaustion rather than exclusively supporting autonomy.

### 3.4 The metric fallacy: measuring access, ignoring cost

An analysis of quality improvement mechanisms within the included studies highlights a potential limitation in current evaluation paradigms, conceptualized herein as the *Metric Fallacy*. The 'Metric Fallacy' is introduced here as an author-proposed critique of existing measurement models based on the synthesized evidence. Analysis of the 35 included studies identifies a significant reliance on two primary measurement modalities: retrospective satisfaction instruments (n = 21), such as the Family Satisfaction in the Intensive Care Unit (FS-ICU) scale, and attendance-based metrics (n = 14) quantifying visitation duration (Table 1). While these tools assess the perceived quality of service and physical access, they generally overlook the surrogate’s internal physiological state. Specifically, 0% of the non-interventional clinical studies in the synthesized cohort incorporated objective biomarkers of stress or real-time cognitive monitoring. This empirical gap suggests that current evaluation frameworks may incorrectly equate high satisfaction and prolonged presence with sustainable engagement. This discrepancy forms the basis of the 'Metric Fallacy,' as the physiological and cognitive 'cost' of engagement to the surrogate remains largely unquantified within existing institutional metrics.

The efficacy of family engagement is frequently evaluated through access duration (visitation hours) and retrospective satisfaction surveys. Literature suggests these indicators may lack the sensitivity required to detect surrogate allostatic load (Fumis et al., 2015; Heyland et al., 2002). This limitation indicates a potential need to revise participation programs to help mitigate unintended psychological morbidity (Rahimi-Bashar et al., 2024).

Relying primarily on satisfaction scores may obscure the physiological toll on families. Extracted data suggest that family members may report high satisfaction based on uninterrupted bedside access, potentially equating physical endurance with optimal patient advocacy (Fumis et al., 2015). While observational studies indicate that institutions often allow families to witness complex clinical interventions (Jabre et al., 2013), this synthesis suggests that this practice may persist partly due to an absence of protocols addressing the surrogate's perceived moral obligation to remain present.

In such environments, clinical practices may inadvertently contribute to surrogate burnout. While prior literature recognizes the depletion of family coping mechanisms (White et al., 2018), the proposed framework questions the tendency to approach familial endurance without monitoring physiological reserve as a safety parameter. The synthesized evidence suggests that this approach may disproportionately shift the responsibility for boundary-setting onto individuals already experiencing significant psychological stress (Cameron et al., 2016).

### 3.5 Titrating family presence for sustainability

To transition toward sustainable engagement, the synthesized evidence supports the conceptualization of a structured approach: the *Clinical Titration of Family Presence*. Situated within the Conservation of Resources (COR) theory, this analysis frames titration not as a restriction of access, but as the structured regulation of engagement intensity aimed at supporting the surrogate’s physiological and psychological capacity. The Clinical Titration of Family Presence is presented as a proposed hypothesis framework for future investigation into sustainable surrogate engagement.

Extracted data suggest that current protocols often approach family presence as a binary variable (present versus absent) (Agård and Lomborg, 2011). The proposed model of clinical titration introduces the principle of *Restorative Dosing*. Literature indicates that a temporary reduction in physical presence can provide a necessary physiological recovery interval (Schmidt and Azoulay, 2013); this synthesis frames the facilitation of this interval as a process of ethical unburdening (Wilson et al., 2019). Because forced separation without adequate psychological support may exacerbate distress (Eftekhari et al., 2025), the clinical titration protocol is designed to function as an authorization for rest. Clinicians validate the family’s presence while reframing continuous vigilance from an obligation to a potential physiological risk, assuming temporary responsibility to help mitigate the surrogate's feelings of abandonment guilt. By structuring these restorative intervals, the intervention aims to support the preservation of cognitive faculties necessary for shared decision-making.

In operationalizing this approach, the framework incorporates data highlighting the importance of structured and continuous communication (Rahimi-Bashar et al., 2022). Therefore, clinical titration is conceptualized as a dynamic and responsive process rather than a static protocol. Aligning with evidence regarding fluctuating family stamina (Xyrichis et al., 2021), this strategy adapts the balance between presence and respite in response to both the patient’s clinical trajectory and the surrogate's physiological reserves.

### 3.6 Beyond titration: deploying a multimodal resilience ecosystem

The synthesized literature emphasizes the importance of physiological recovery (Schmidt and Azoulay, 2013). To operationalize this, the synthesis introduces the concept of *Active Restoration*, incorporating evidence-based non-pharmacological modalities to help mitigate psychological distress (Jafarabadi et al., 2020; Vahedian-Azimi et al., 2014).

The thematic synthesis informed the development of the *Pan-Dimensional Matrix for Surrogate Resilience*, based on Stevan E. Hobfoll’s Conservation of Resources (COR) theory (Hobfoll, 2001). As detailed in Table 2, the proposed mechanisms and metrics are categorized based on their evidentiary status—ranging from direct empirical support to extrapolations from general stress models or newly proposed concepts for future clinical validation. This matrix (Table 2) conceptualizes the Intensive Care Unit as an ecosystem encompassing physiological, cognitive, psycho-spiritual, social, and technological domains aimed at preserving surrogate resources. Rather than viewing psychosocial support solely as adjunctive, the matrix conceptualizes these interventions as functional supports, utilizing documented mechanisms (Ayenew et al., 2025; Checa-Checa et al., 2025; Lucchini et al., 2025; Shirasaki et al., 2024) across five interconnected dimensions:

**Table 2:** The pan-dimensional matrix for surrogate resilience: operationalizing the clinical titration of presence via a bio-psycho-social-technological ecosystem (Grounded in conservation of resources theory).

**Dimension**	**Specific intervention strategy**	**Targeted deficit / neurobiological mechanism**	**Precision outcome metrics**
**1. Physiological & Environmental Infrastructure**	**Chronobiological Restoration Zones** (**Circadian-Aligned Architecture**)	**Target:** Suprachiasmatic Nucleus Desynchronization. **Mechanism:** Utilization of dynamic lighting and acoustic isolation to entrain circadian rhythms, facilitate REM rebound, and clearance of hypothesized adenosine load [Proposed], thereby preventing " sleep-related cognitive disruption [Proposed]" in surrogates.	• **Surrogate Actigraphy:** Sleep Fragmentation Index (SFI) & Total Sleep Time (TST). • **Cognitive Vigilance:** Psychomotor Vigilance Task (PVT) scores. • **Delirium Incidence:** CAM-ICU (Family Adaptation; Proposed Adaptation).
	**Metabolic & Hydration Optimization Bundles**	**Target**: Potential metabolic influence on executive function [Extrapolated]. **Mechanism:** Proactive delivery of high-protein/complex-carb nutrition to stabilize glycemic variability and prevent "Decision Fatigue" caused by metabolic depletion during prolonged bedside vigils.	• **Glycemic Stability:** Incidence of reported hypoglycemic/vasovagal events. • **Physical Stamina:** Borg Rating of Perceived Exertion (RPE). • **Nutritional Adherence:** Daily caloric intake vs. estimated requirement.
	**Biophilic & Salutogenic Design Elements**	**Target:** Sympathetic Nervous System Hyperarousal. **Mechanism:** Visual integration of nature (Healing Gardens) to trigger parasympathetic activation and lower systemic cortisol via evolutionary "Biophilia" pathways.	• **Stress Biomarkers:** Salivary Cortisol Area Under the Curve (AUC). • **Autonomic Response:** Heart Rate Variability (HRV) coherence. • **Psychological Restoration:** Perceived Restorativeness Scale (PRS).
	**Dignity & Hygiene Preservation Facilities**	**Target:** Somatic Discomfort & Loss of Selfhood. **Mechanism:** Provision of private hygiene suites to mitigate the physical degradation associated with "rough sleeping" in waiting rooms, preserving the surrogate's sense of agency and dignity.	• **Dignity Metrics:** Patient Dignity Inventory (PDI-Family Version; Proposed Adaptation). • **Subjective Well-being:** Visual Analog Scale (VAS) for physical comfort. • **Facility Utilization:** Rate of shower/hygiene suite usage per admission.
**2. Cognitive & Information Prosthetics**	**Structured Cognitive Scaffolding** (**VALUE Protocol**)	**Target:** Cognitive Overload & Information Asymmetry. **Mechanism:** Utilization of the VALUE mnemonic to structure complex prognostic data, reducing the "allostatic load" on the surrogate's prefrontal cortex during high-stakes decision-making.	• **Prognostic Concordance:** Agreement rate between clinician and family understanding of prognosis. • **Satisfaction:** FS-ICU 24R (Decision-Making Subscale). • **Comprehension:** Quality of Communication (QOC) Scale.
	**Visual Decision Algorithms & Infographics**	**Target:** Probabilistic Reasoning Failure. **Mechanism:** Mapping disease trajectories visually to externalize working memory demands, allowing surrogates to process stochastic data without "Analysis Paralysis."	• **Decisional Conflict:** Decisional Conflict Scale (DCS) scores. • **Decision Regret:** Decision Regret Scale (DRS) at 3 months. • **Consensus Velocity:** Time elapsed from prognosis to Goals of Care (GoC) consensus.
	**Health Literacy Translation** (**Lay Language Glossaries**)	**Target:** Semantic Anxiety ("Medicalese"). **Mechanism:** Bridging the linguistic gap to reduce anxiety stemming from terminological ambiguity, thereby empowering the surrogate's "Health Literacy."	• **Literacy Assessment:** Short Assessment of Health Literacy (SAHL-ICU). • **Anxiety Reduction:** State-Trait Anxiety Inventory (STAI-State). • **Clarification Index:** Rate of nursing interruptions for terminology clarification.
**3. Psycho-Spiritual Scaffolding**	**Anticipatory Grief & Bereavement Preparation**	**Target:** Complicated Grief & Traumatic Stress. **Mechanism:** Preemptive psychological inoculation to normalize the grieving process, preventing the pathological encoding of traumatic memories (PTSD precursor).	• **Grief Trajectory:** Inventory of Complicated Grief (ICG) at 6 months. • **Trauma Symptoms:** Impact of Event Scale-Revised (IES-R). • **Family Function:** Family Adaptation and Cohesion Evaluation Scale (FACES IV).
	**Crisis De-escalation Protocol** (**Code Lavender**)	**Target:** Acute Emotional Dysregulation. **Mechanism:** Rapid-response holistic intervention (bio-psycho-spiritual) to stabilize the "Emotional acute emotional dysregulation [Extrapolated]" following bad news or traumatic witnessing.	• **Acute Distress:** Subjective Units of Distress (SUDS) pre/post intervention. • **Staff Morale:** Moral Distress Scale-Revised (MDS-R) for clinicians. • **Stabilization Time:** Minutes required to return to functional baseline.
	**Existential Anchoring** (**Logotherapy/Meaning-Making**)	**Target:** Existential Nihilism & Spiritual Distress. **Mechanism:** Logotherapy-based consults to help surrogates construe meaning from suffering, buffering against the "Crisis of Purpose."	• **Spiritual Well-being:** FACIT-Sp (Functional Assessment of Chronic Illness Therapy-Spiritual). • **Meaning:** Meaning in Life Questionnaire (MLQ). • **Hope:** Herth Hope Index (HHI).
	**Digital Legacy & Memory Construction**	**Target:** Fear of Erasure & Disconnection. **Mechanism:** Assisted curation of the patient's narrative (voice/photos) to foster a "Continuing Bond," facilitating healthier closure and bereavement.	• **Bereavement Risk:** Bereavement Risk Assessment Tool (BRAT; Proposed Adaptation). • **Quality of Death:** Quality of Dying and Death (QODD) - Family perception. • **Legacy Engagement:** Frequency of interaction with legacy artifacts post-discharge.
**4. Social & Logistical Architecture**	**Financial Toxicity Screening & Mitigation**	**Target:** Economic Resource Depletion. **Mechanism:** Proactive identification of "Financial Toxicity" (lost wages, lodging costs) to prevent the secondary trauma of economic collapse.	• **Economic Burden:** Comprehensive Score for Financial Toxicity (COST-FAC). • **Intervention Speed:** Time to Social Work referral. • **Resource Utilization:** Out-of-pocket expenditure vs. available aid.
	**Rotational Vigilance Protocol** (**Shift-Work Model**)	**Target:** Single-Surrogate Burnout Syndrome. **Mechanism:** Formalizing a "Relay Model" of caregiving to distribute the vigilance burden across the kinship network, preserving the primary decision-maker's reserve.	• **Caregiver Burden:** Caregiver Burden Inventory (CBI). • **Attrition Rate:** Rate of single-surrogate exhaustion/dropout. • **Burden Severity:** Zarit Burden Interview (ZBI) - Short Form.
	**Navigator / Liaison Nurse Role**	**Target:** Logistical Disorientation. **Mechanism:** Decoupling clinical updates from administrative logistics, reducing the "Cognitive Friction" of navigating hospital bureaucracy.	• **Administrative Friction:** Administrative Burden Index (ABI). • **System Navigation:** Family Satisfaction with Decision Making (FS-ICU-DM). • **Efficiency:** Reduction in non-clinical calls to bedside nurses.
	**Peer Mentorship Programs**	**Target:** Social Isolation & Hopelessness. **Mechanism:** Facilitating "Vicarious Resilience" by connecting current families with ICU survivors, providing a roadmap for survival.	• **Social Support:** Social Support Effectiveness Questionnaire (SSEQ). • **Psychological Growth:** Post-Traumatic Growth Inventory (PTGI). • **Isolation:** UCLA Loneliness Scale.
**5. Technological & Predictive Connectivity**	**AI-Driven Risk Stratification** (**Predictive Analytics**)	**Target:** Undetected Compulsive Hyper-engagement. **Mechanism:** Utilizing Natural Language Processing (NLP) and behavioral patterns to algorithmically predict families at high risk of PICS-F before collapse occurs.	• **Algorithm Accuracy:** Sensitivity/Specificity of PICS-F prediction models. • **Early Warning:** Mean time from admission to high-risk flag generation. • **Preventative Efficacy:** Number of "Rescue Interventions" triggered by AI.
	**Tele-Presence & EHR Integration** (**OpenNotes**)	**Target:** Anxiety of Uncertainty (Separation Anxiety). **Mechanism:** Providing high-fidelity virtual presence and transparent EHR access to mitigate the "Fear of Missing Out" (FOMO) on clinical changes during rest periods.	• **Digital Health Literacy:** eHEALS (eHealth Literacy Scale). • **Anxiety Correlation:** Frequency of EHR log-ins vs. STAI scores. • **Remote Engagement:** Number of "Virtual Bedside Rounds" attended.

This matrix delineates the specific interventions deployed as **"Restorative Doses"** during the **Clinical Titration of Family Presence**. Grounded in the **Conservation of Resources** (**COR**) **theory**, these *Functional Prosthetics* aim to prevent allostatic depletion and circumvent the **Metric Fallacy** by utilizing precision objective metrics. Ultimately, this structural scaffolding facilitates the transition from a state of *Compulsive Hyper-engagement* to the emergence of a resilient, **Capacitated Partner**.

**Physiological & Environmental Infrastructure:** This tier focuses on chronobiological support and metabolic optimization, aiming to mitigate sleep deprivation delirium and decision fatigue (Ashghab et al., 2024; Hamilton et al., 2025).

**Cognitive & Information Prosthetics:** To address information asymmetry, this dimension utilizes structured communication frameworks (Hwang et al., 2025; Bhatt et al., 2025; Lautrette et al., 2007; Vahedian-Azimi et al., 2016) and visual decision aids [35] to potentially reduce cognitive load.

**Psycho-Spiritual Scaffolding:** This tier incorporates anticipatory grief protocols and existential support to assist in processing psychological trauma (Boehm et al., 2020; Stone, 2018; Bashar et al., 2018; Kang et al., 2019; Vahedian-Azimi et al., 2023; Farzanegan et al., 2021).

**Social & Logistical Architecture:** This dimension includes financial screening and rotational visitation protocols, emphasizing the role of structural support in resilience (Khandelwal et al., 2025).

**Technological & Predictive Connectivity:** This involves the use of AI-assisted risk stratification and tele-presence technologies to monitor potential burnout and provide connection during restorative intervals (Pell et al., 2015; Rosenthal et al., 2024; Arabfard et al., 2025; Pandian et al., 2026).

By integrating these five domains, supported by broader resilience literature (Alhani et al., 2022; Cai et al., 2026), this framework aims to transition surrogates from susceptible observers to *Capacitated Partners*.

**Fig. 1:**
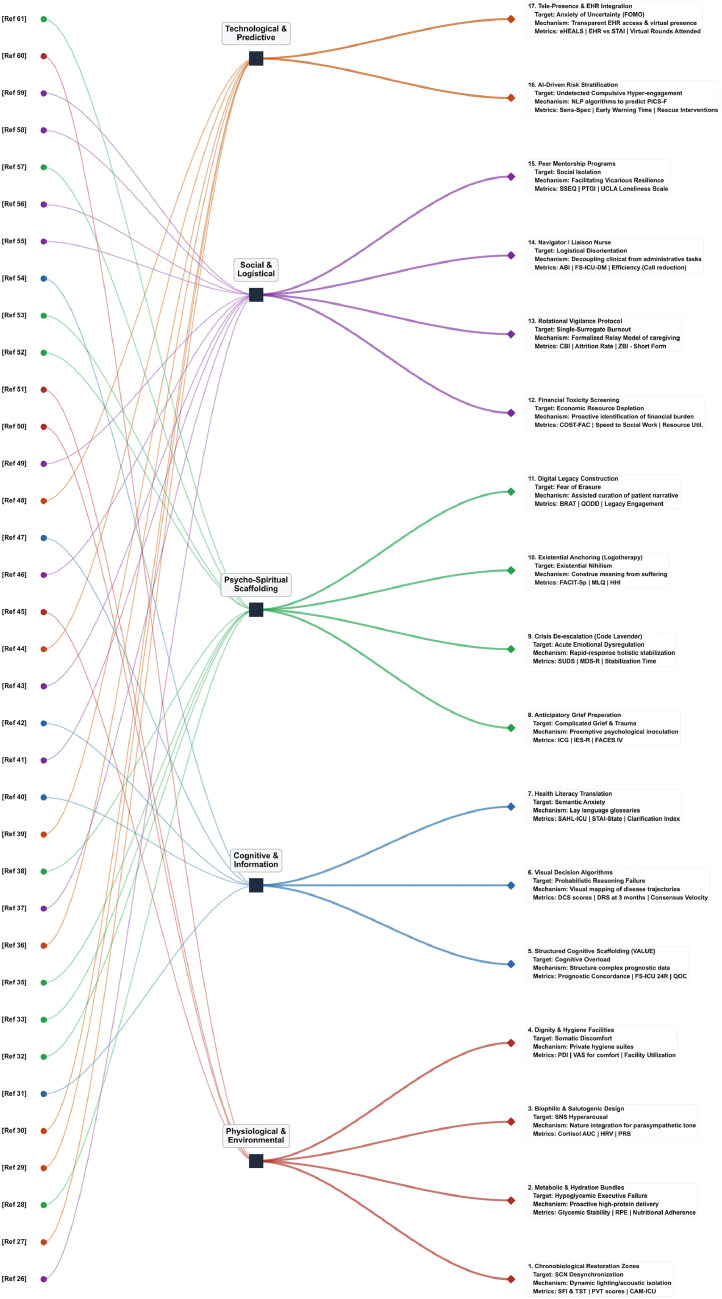
The cascading epistemological network: a conceptual schematic mapping the translation of empirical evidence into clinical interventions within the RESTORE framework. This three-tiered bipartite network serves as a heuristic visual metaphor for the evidence synthesis process. The schematic illustrates the conceptual transition from the extracted empirical data to theoretical dimensions, and subsequently to the proposed clinical interventions. *(Left Tier - The Evidence Base):* Individual nodes represent the 35 included narrative synthesis articles, spatially distributed to optimize visualization. *(Central Axis - The Conceptual Scaffolding):* Convergent trajectories map the empirical literature onto the five foundational domains of the Conservation of Resources (COR) theory. The spatial positioning of these central domains is heuristically organized to represent their relationship with the respective downstream interventions. *(Right Tier - The Clinical Matrix):* The theoretical domains connect to 17 proposed functional interventions. Each intervention block is delineated by its primary neurobiological or psychological target, its proposed active mechanism, and the specific metrics intended to quantify surrogate resilience.

To visualize the translation of empirical evidence (Table 1) into the components of the Resilience Ecosystem for Surrogate Titration, Overload Recovery, and Engagement (RESTORE) framework (Table 2), a bipartite network is presented (Fig. 1). This matrix maps the 35 synthesis articles to their corresponding targets, mechanisms, and metrics across the five domains.

### 3.7 The paradox of autonomy and institutional safeguards

Thematic appraisal of the literature requires examination of the ethical considerations regarding the regulation of family presence, particularly concerns related to paternalism. The proposed framework contextualizes the ethical basis of clinical titration within a concept termed the *Paradox of Agency*.

Evidence suggests that functional autonomy relies on cognitive capacity. While observational data indicate that a surrogate under sustained allostatic load may retain legal decisional authority (Beesley et al., 2018), this may occur alongside a measurable decline in deliberative capacity (White et al., 2018). Based on these data, this synthesis hypothesizes that in states of acute cognitive fatigue, unstructured continuous access represents a plausible risk to the surrogate's functional capacity. Therefore, interventions designed to restore physiological reserve may serve to support, rather than override, surrogate autonomy.

To guide the appropriate application of this framework, specific institutional exclusion criteria are proposed. Consistent with guidelines stating that family access should not be restricted for logistical reasons (Davidson et al., 2017), clinical titration is indicated exclusively in response to the physiological fatigue of the surrogate. Utilizing this approach for institutional convenience, staff workload management, or spatial constraints is outside its intended ethical scope. To preserve the ethical integrity of the framework, clinical titration must be voluntary, personalized, and culturally sensitive. The process is contingent upon informed consent and must remain strictly non-punitive. Clinical titration should never be utilized to address institutional workflow, staffing requirements, or spatial constraints. Furthermore, the operationalization of this model necessitates the consideration of patient preferences, surrogate legal authority, and potential family conflicts. To support equity and data privacy, restorative interventions should be accessible to all family units, maintaining strict confidentiality regarding surrogate health indicators and engagement patterns.

While existing literature emphasizes the importance of addressing cultural dynamics within the Intensive Care Unit (Vahedian-Azimi, 2025), the synthesis highlights the need to differentiate between physiological fatigue and cultural practices. In various demographic contexts, continuous presence serves as a cultural or relational expectation (Giannini et al., 2013). Therefore, the data suggest that intense familial involvement, provided it remains physiologically sustainable (Day et al., 2013) and culturally congruent, should be distinguished from the state conceptualized as *Compulsive Hyper-engagement*.

### 3.8 The stakeholder triad: collateral implications for patient and clinician

The thematic synthesis suggests that the implications of this framework extend to the broader Intensive Care Unit stakeholder triad. While the preceding sections focused on surrogate capacity, the extracted data also indicate relevant implications for the patient and the healthcare professional. From the patient perspective, literature describes an exhausted surrogate as a potentially compromised advocate (White et al., 2018); accordingly, this analysis suggests that permitting allostatic exhaustion to negatively impact executive function may inadvertently affect patient safety during shared decision-making.

From the clinician perspective, evidence indicates that observing family exhaustion without intervention can contribute to moral distress (Agård and Lomborg, 2011; Khaghanizadeh et al., 2023). Included studies report that clinicians may experience distress when witnessing psychological strain in families, particularly when procedural policies limit their ability to intervene (Vahedian-Azimi et al., 2019). Empowering the interdisciplinary team to utilize the clinical titration of presence may allow clinicians to take a more active role in supporting the family unit. The proposed framework suggests that this structured regulation aligns with the bioethical principle of non-maleficence (Wilson et al., 2019).

**Fig. 2:**
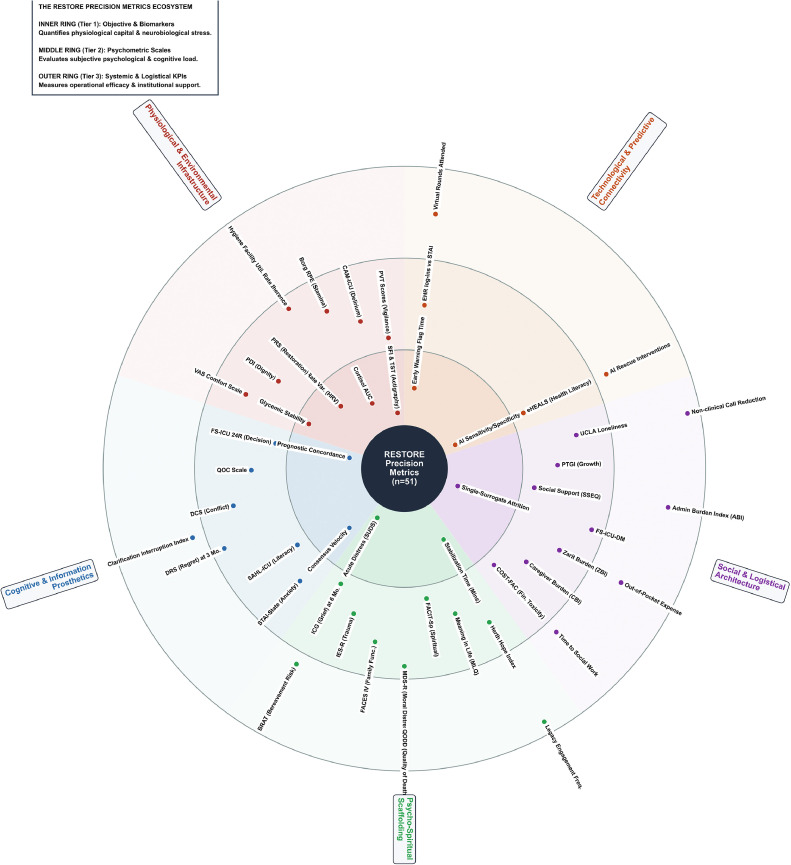
**The multi-axial evaluation stratogram: a conceptual radial topology for evaluating surrogate capacity.** This radial stratogram is a conceptual schematic visualizing the 51 proposed outcome metrics delineated within the RESTORE framework, organizing them into a multidimensional heuristic structure. This topology aims to address the aforementioned Metric Fallacy by categorizing measurements across three distinct modalities to support a triangulated evaluation approach. *(Tier 1 – Core):* The innermost ring maps objective physiological and neurobiological biomarkers proposed to quantify indicators of the surrogate's acute allostatic load. *(Tier 2 - Intermediate Ring):* The middle ring encompasses standardized, psychometrically validated clinical instruments evaluating subjective cognitive load. *(Tier 3 - Outer Ring):* The peripheral ring identifies systemic and logistical key performance indicators (KPIs) to assess the operational characteristics of the Intensive Care Unit environment.

To facilitate future empirical validation of the Resilience Ecosystem for Surrogate Titration, Overload Recovery, and Engagement (RESTORE) framework, 51 proposed outcome metrics have been organized into a multidimensional radial topology (Fig. 2). This *Multi-Axial Precision Evaluation Stratogram* aims to address the aforementioned evaluation limitations by categorizing endpoints into three measurement modalities: objective neurobiological biomarkers, validated psychometric scales, and systemic logistical indicators. This tripartite structure provides a multi-level blueprint for evaluation.

## 4 Discussion

The RESTORE (Resilience Ecosystem for Surrogate Titration, Overload Recovery, and Engagement) synthesis evaluates family integration in the intensive care unit. This review approaches surrogate presence as both an ethical issue and a physical demand. Findings suggest that unregulated access without structural support may contribute to "Compulsive Hyper-engagement." Using Hobfoll’s Conservation of Resources (COR) theory (Hobfoll, 1989), the Pan-Dimensional Matrix and the Clinical Titration framework provide a structured approach. Rather than a confirmed clinical intervention, the Clinical Titration model is presented here as a conceptual framework proposed for future feasibility and efficacy testing. These tools address evaluation metrics that may mistakenly equate a surrogate's physical endurance with optimal care.

### 4.1 Conceptual congruence: alignment with concordant literature

The proposed framework aligns with recent critical care literature on the psychological demands of surrogate engagement. Our findings regarding physical stress and cognitive changes align with studies showing that severe Intensive Care Unit sleep disruption may impair decision-making (Pignatiello et al., 2023; Huang et al., 2024). This agreement highlights the importance of measuring objective physical signs alongside subjective surveys. Furthermore, linking Compulsive Hyper-engagement to Post-Intensive Care Syndrome-Family (PICS-F) is consistent with longitudinal cohorts observing increased Post-Traumatic Stress Disorder (PTSD) and anxiety among families with prolonged bedside presence (Ayenew et al., 2025; Putowski et al., 2023).

The proposed Active Restoration interventions are supported by interventional trials exploring the role of environmental design and sleep support in reducing physical fatigue (Knauert et al., 2023; Gunnlaugsdóttir et al., 2024). Additionally, the Clinical Titration model addresses the moral distress experienced by clinicians who witness surrogate exhaustion, a phenomenon supported by qualitative analyses of Intensive Care Unit nurses (Korsah et al., 2026; de Beer et al., 2026). Finally, the technological connectivity dimension aligns with predictive analytics research. This includes using AI risk assessment and tele-presence to reduce anxiety while surrogates rest (Michalsen et al., 2019; Teixeira and Rosa, 2025). This alignment across the literature suggests that evaluating resilience through physical and mental capacity, rather than just visitation duration, supports the use of active support frameworks.

### 4.2 Epistemological divergence: deconstructing discordant literature

Conversely, this framework differs from studies that view unrestricted family access as the primary indicator of patient-centered care. Some studies suggest that regulating presence may increase surrogate anxiety and limit autonomy (Rosa et al., 2025; Milner and Marmo, 2026). Other studies frame continuous bedside vigilance as a cultural coping mechanism linked to high satisfaction, rather than as Compulsive Hyper-engagement (Wu et al., 2022; BULUT et al., 2025). Certain operational models may also treat surrogates as extensions of the clinical team, requiring their continuous presence (Halm et al., 2024; Lassi et al., 2025).

This synthesis suggests that these divergent findings may stem from differences in evaluation methodologies rather than contradictory biological mechanisms. Studies supporting unrestricted presence frequently utilize retrospective satisfaction surveys. These surveys may fail to capture acute physical stress or real-time cognitive decline (Bhatt et al., 2025; Huang et al., 2025). Additionally, bioethical frameworks cautioning against paternalistic interventions may prioritize theoretical autonomy but may overlook how extreme stress impairs actual decision-making capacity (Lim et al., 2025; Abdalla et al., 2025). Consequently, the present findings diverge because the Conservation of Resources (COR)-based framework focuses on sustaining the surrogate's physical and mental capacity, while contrasting literature focuses primarily on maintaining physical access.

### 4.3 Synthesis of the evidence: bridging the dichotomy

**Fig. 3:**
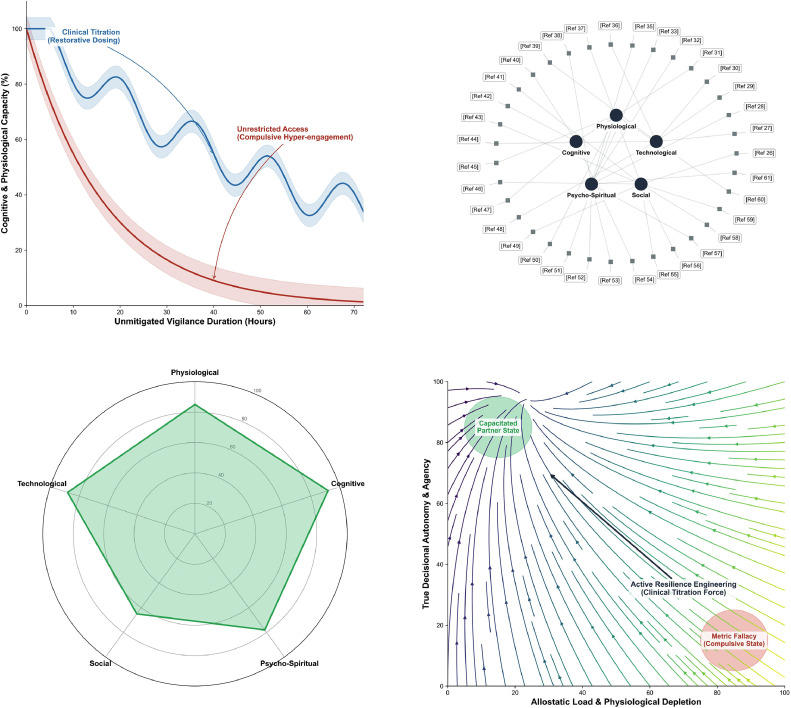
The RESTORE epistemological state-space matrix: a conceptual schematic and multidimensional evidence mapping. This four-panel composite visualization utilizes visual metaphors to integrate the core arguments and synthesized findings of the RESTORE framework, conceptualizing the transition from physiological depletion to sustained capacity. *(A) The Allostatic Depletion Trajectory (Conceptual Paradigm):* A conceptual trajectory comparing two hypothesized clinical models. The red curve illustrates the potential decline in surrogate capacity under continuous visitation policies, whereas the blue curve represents the proposed Clinical Titration approach. *(B) The Evidence Topology & Dimensional Mapping:* A heuristic chord diagram mapping the 35 included empirical articles to the five central domains of the COR theory. *(C) The Pan-Dimensional Radar Density:* A conceptual polar area chart reflecting the relative thematic concentration of targeted functional interventions extracted from the literature across the five specified domains. *(D) The Phase-Space Shift (Heuristic Representation):* A conceptual state-space representation utilizing vector-field motifs to illustrate the hypothesized psychological transition of the ICU surrogate from a high-load, low-agency state toward a stabilized, higher-agency equilibrium.

Synthesizing this literature reveals a tension between strictly regulated and entirely unrestricted Intensive Care Unit access. While some literature highlights the psychological demands of the open model, contrasting studies caution against the potential paternalism of restricted models. The Pan-Dimensional Matrix and the concept of Clinical Titration aim to bridge these perspectives. In this context, 'restorative dosing' and 'titration' are utilized as theoretical metaphors to describe a hypothesized model of structured engagement. This framework frames temporary rest not as a restriction of rights, but as a necessary health intervention ("restorative dosing"). This approach supports autonomy while protecting the surrogate's mental health. It shifts the focus from managing physical presence to ensuring sustainable engagement. To visualize this conceptualization, the trajectory and empirical structure of this synthesis are mapped in Fig. 3. This multi-dimensional Epistemological State-Space Matrix visually represents the physical stress associated with continuous access, the distribution of the synthesized evidence base, the integration of proposed interventions, and the hypothesized transition of the surrogate toward capable shared decision-making.

### 4.4 Implications for clinical practice

Applying this synthesis to practice suggests a shift in critical care nursing. Interdisciplinary teams may benefit from transitioning from simply allowing access to actively structuring surrogate resilience. In clinical practice, this involves assessing family members for signs of acute mental fatigue, such as severe sleep loss or confusion, which differ from normal grief. For example, if a surrogate remains at the bedside for 48 consecutive hours and begins struggling to comprehend basic medical updates, the clinician can intervene. Using the titration framework, the nurse can explicitly authorize a structured rest period, temporarily assuming the monitoring role to relieve the surrogate's sense of duty. Integrating the Clinical Titration of Presence into standard nursing workflows provides a method for structured conversations. It allows clinicians to formally encourage rest, helping families avoid feelings of guilt or abandonment. The operationalization of the Resilience Ecosystem for Surrogate Titration, Overload Recovery, and Engagement (RESTORE) framework necessitates a transition from theoretical constructs to pragmatic nursing workflows. In clinical practice, this is achieved through structured nursing actions, such as conducting routine surrogate fatigue and support-needs assessments and establishing formalized communication plans to maintain family connectivity during authorized rest intervals. Furthermore, the integration of 'Social & Logistical Architecture' involves providing liaison or navigator support, ensuring access to designated quiet areas, and facilitating timely referrals to social work and chaplaincy services. By documenting these specific support requirements within the clinical record, nursing teams can transition the intensive care unit into a structured ecosystem that actively sustains surrogate capacity. This approach helps ensure that families remain capable partners in shared decision-making.

### 4.5 Strengths and limitations

A primary strength of this synthesis is its structured methodological approach. By integrating the Scale for the Assessment of Narrative Review Articles (SANRA) and Joanna Briggs Institute (JBI) methodologies, the evidence appraisal aimed to minimize selection biases often associated with traditional narrative reviews. Additionally, the automated extraction and documentation of metadata supported data integrity when managing large datasets, contributing to the computational reproducibility of the synthesis. However, certain limitations must be acknowledged. The inclusion criteria were restricted to English-language publications; while intended to capture a broad range of high-impact literature, this parameter may exclude culturally specific perspectives on family engagement documented in localized languages. Furthermore, the conceptual and thematic focus of this review precluded a quantitative meta-analysis. Future empirical research is needed to quantitatively evaluate the specific interventions outlined within the *Pan-Dimensional Matrix* across diverse Intensive Care Unit demographics.

## 5 Conclusion

The transition toward open-access Intensive Care Units represents a significant evolution and development in critical care practice; however, the Resilience Ecosystem for Surrogate Titration, Overload Recovery, and Engagement (RESTORE) synthesis identifies a plausible gap in current operationalization, suggesting the hypothesis that unrestricted access may benefit from the concurrent implementation of structural safeguards to address potential physiological demands on surrogates. By examining the limitations of current evaluation metrics, which may frequently equate prolonged physical presence with optimal patient advocacy, this review highlights the potential psychological and physiological effects and risks associated with Compulsive Hyper-engagement. The synthesized evidence suggests a plausible association between prolonged exposure to unmitigated allostatic load and acute cognitive alterations, which may inadvertently compromise surrogate autonomy and affect functional capacity. To address these considerations and fill this gap, this study proposes an evidence-based framework aimed at supporting Active Resilience Engineering within the Intensive Care Unit, presented as a conceptual model for future empirical investigation into its feasibility, acceptability, equity, safety, and clinical outcomes.

Through the operationalization of the *Pan-Dimensional Matrix for Surrogate Resilience* and the *Clinical Titration of Presence*, the proposed framework conceptualizes family support as a structured system of functional interventions. This model suggests that temporary, clinician-guided respite, conceptualized as ethical unburdening, may support rather than restrict surrogate agency. By providing a restorative interval, this approach aims to preserve the cognitive capacity necessary for participation in shared decision-making.

Ultimately, sustainable family integration may require clinical frameworks to incorporate surrogate physiological and psychological capacity as a standard metric of safety. Comprehensive patient-centered care may benefit from protocols designed to assist families in managing the perceived obligation of continuous vigilance. By structurally regulating the intensity of presence, interdisciplinary teams can aim to foster a supportive ecosystem that sustains surrogate resources. This approach seeks to assist families in maintaining their role as *Capacitated Partners* throughout the trajectory of critical illness.

## Ethics approval and consent to participate

Not applicable. As this study constitutes a systematic synthesis of existing published literature to construct a theoretical framework, it does not involve primary data collection, human participants, animal subjects, or clinical interventions. Consequently, institutional ethical approval and informed consent are not required.

## Consent for publication

Not applicable. This manuscript does not contain identifiable personal data, individual clinical images, or private health information; therefore, consent for publication is not mandated.

## Availability of data and materials

All empirical data and conceptual matrices generated or analyzed during this study are included in this published article. This specifically encompasses the documented Scale for the Assessment of Narrative Review Articles (SANRA) compliance checklist and the exhaustive multi-database search algorithms.

## Funding

This research received no specific grant from any funding agency in the public, commercial, or not-for-profit sectors.

## Declaration of generative AI and AI-assisted technologies in the writing process

The authors acknowledge the utilization of generative artificial intelligence (specifically, Gemini) strictly for advanced linguistic refinement, structural formatting assistance, and the technical rendering of the conceptual visual abstract. No AI technologies were employed to generate the foundational scientific hypotheses, interpret clinical data, or formulate the intellectual medical constructs within this manuscript. The authors maintain ultimate oversight and assume full responsibility and accountability for all content herein.

## CRediT authorship contribution statement

**Ali Bahramifar:** Writing – review & editing, Writing – original draft, Conceptualization. **Amir Vahedian-Azimi:** Writing – review & editing, Writing – original draft, Visualization, Validation, Supervision, Software, Methodology, Conceptualization.

## Declaration of competing interest

The authors declare the absence of any financial or non-financial competing interests regarding the publication of this paper.

## Appendix Supplementary materials

Image, application 1

Image, application 2

Figure S1. Resource Dynamics within the RESTORE Framework This conceptual schematic illustrates the application of the Conservation of Resources (COR) theory. It contrasts the hypothesized trajectory of an unmitigated resource loss spiral (characteristic of continuous, unstructured vigilance) against the proposed restorative gain spiral associated with the clinical titration of presence. Image, image 3 dummy alt text

Figure S2. The Methodological Contribution and Appraisal Matrix This bubble matrix visualizes the intermediate translational pathway from primary evidence extraction to the Resilience Ecosystem for Surrogate Titration, Overload Recovery, and Engagement (RESTORE) framework. The Y-axis represents the five deductive COR domains, while the X-axis categorizes the inductive sources by study design. Bubble size corresponds to the volume of thematic codes extracted, and color opacity reflects the average methodological weighting derived from the Joana Briggs Institute (JBI) quality appraisal, illustrating how each source type influenced each specific domain. Image, image 4 dummy alt text

Image, application 5
