# Supplementary material for: Surrogate resilience and clinical titration of presence in the open intensive care unit: a systematic narrative synthesis
Source: Int J Nurs Stud Adv. 2026 Jun 8;11:100583. doi: 10.1016/j.ijnsa.2026.100583 (PMC13279037; doi:10.1016/j.ijnsa.2026.100583)
Supplement: Supplementary file 1 [file mmc1.docx]

# Electronic Supplementary Materials 1

# ESM S1. SANRA (Scale for the Assessment of Narrative Review Articles) Compliance Checklist

**Manuscript Title: Surrogate Resilience and Clinical Titration of Presence in the Open Intensive Care Unit: A Systematic Narrative Synthesis**

**Author:** Professor Amir Vahedian-Azimi

This document serves as the formal methodological declaration, confirming that the aforementioned manuscript has been architected in strict, uncompromising adherence to the Scale for the Assessment of Narrative Review Articles (SANRA) guidelines (Baethge et al., 2019). The following matrix delineates the alignment of the manuscript’s structural and epistemological components with the 6 core Scale for the Assessment of Narrative Review Articles (SANRA) criteria, explicitly justifying the assignment of the maximum score (2) across all domains.

# ESM S1. SANRA (Scale for the Assessment of Narrative Review Articles) Compliance Checklist

| **Item** | **SANRA Criterion** | **Score** | **Manuscript Alignment / Location** |
| --- | --- | --- | --- |
| 1 | **Justification of the article’s importance for the readership** | **2** | The critical necessity of this synthesis is explicitly architected in the 'Introduction'. The manuscript transcends standard descriptive paradigms by deconstructing the 'Metric Fallacy' and introducing the novel 'Compulsive Hyper-engagement' phenomenon. It convincingly argues that unregulated ICU access precipitates acute cognitive decompensation in surrogates, thus justifying the urgent need for a paradigm shift (See: **Introduction section**). |
| 2 | **Statement of concrete aims or formulation of questions** | **2** | The epistemological objective is stated with absolute precision at the culmination of the Introduction: '...this narrative review aims to systematically synthesize the disparate literature regarding surrogate psychological burden... By adopting Stevan E. Hobfoll’s Conservation of Resources (COR) theory as the overarching theoretical lens, this review seeks to map the empirical evidence onto a novel, five-dimensional Pan-Dimensional Matrix...' (See: **End of Introduction**). |
| 3 | **Description of the literature search** | **2** | The search architecture is exceptionally rigorous, surpassing standard narrative review protocols. Detailed in 'Search Strategy and Information Sources', the methodology specifies a multi-database search (PubMed, Scopus, WoS) utilizing custom Python scripts and APIs for extraction. The exact temporal parameters (up to Feb 24, 2026), a four-dimensional Boolean search matrix, and snowballing techniques are explicitly documented (See: **Methods section and Supplementary Material S2**). |
| 4 | **Referencing** | **2** | Referencing is exhaustively complete and precise. All key declarative statements, ranging from neurobiological mechanisms of allostatic overload to ethical critiques of visitation guidelines, are fortified by 79 highly curated citations. The manuscript meticulously avoids 'orphan claims', ensuring that every conceptual argument is anchored in empirical or theoretical literature (See: **Throughout the manuscript**). |
| 5 | **Scientific reasoning (Use of evidence)** | **2** | Scientific reasoning is fundamentally inductive-deductive and theoretically anchored. The synthesis utilizes Hobfoll's Conservation of Resources (COR) theory to systematically categorize evidence. It critically juxtaposes concordant and discordant literature ('Epistemological Divergence'), identifying methodological artifacts in opposing studies rather than merely listing conflicting results. The evidence translates seamlessly into clinical prosthetics (See: **Results and Discussion**). |
| 6 | **Appropriate presentation of data** | **2** | Data presentation is highly transparent and methodologically auditable. The manuscript explicitly details the selection of a final cohort of 35 core articles. Crucially, the epistemological characteristics, specific study designs, geographic contexts, and conceptual mappings to the COR theory for all 35 studies are meticulously presented in a dedicated matrix ('Table 2'). The PRISMA flow numerics are integrated into the text with exact attrition figures (See: **Methods and Table 2**). |

**Conclusion & Formal Declaration:** Based on the rigorous application of the SANRA methodology, the present narrative synthesis achieves a maximum Cumulative Score of 12/12. This verifies its uncompromising structural integrity, methodological transparency, and readiness for high-impact peer review.
