# Supplementary material for: Surrogate resilience and clinical titration of presence in the open intensive care unit: a systematic narrative synthesis
Source: Int J Nurs Stud Adv. 2026 Jun 8;11:100583. doi: 10.1016/j.ijnsa.2026.100583 (PMC13279037; doi:10.1016/j.ijnsa.2026.100583)
Supplement: Supplementary file 2 [file mmc2.docx]

**Electronic Supplementary Materials 2**

**Search Strategy Protocol (the Joanna Briggs Institute (JBI)** **Standards)**

**Review Title:** Surrogate Resilience and Clinical Titration of Presence in the Open intensive care unit: The RESTORE Narrative Synthesis

**Search Period:** From inception to February 24, 2026.

**Methodology:** A bifurcated three-step search strategy was executed according to the Joanna Briggs Institute (JBI) methodology for narrative and scoping reviews to ensure both breadth of evidence and specificity of the proposed framework.

**1. PubMed (via National Library of Medicine - NLM)**

- **Search Syntax: n = 1108**

(("Intensive Care Units"[MeSH Terms] OR "Critical Care"[MeSH Terms] OR "Intensive Care"[Title/Abstract] OR "ICU"[Title/Abstract])) AND (("Family"[MeSH Terms] OR "Caregivers"[MeSH Terms] OR "Surrogate Decision Making"[MeSH Terms] OR "Family Presence"[Title/Abstract] OR "Family Visitation"[Title/Abstract])) AND (("Burnout, Psychological"[MeSH Terms] OR "Sleep Deprivation"[MeSH Terms] OR "Vigilance"[Title/Abstract] OR "Caregiver Burden"[Title/Abstract] OR "Anxiety"[Title/Abstract] OR "Allostatic Load"[Title/Abstract] OR "PICS-F"[Title/Abstract] OR "Post-Intensive Care Syndrome-Family"[Title/Abstract]) OR ("Compulsive Hyper-engagement"[Title/Abstract] OR "Clinical Titration"[Title/Abstract] OR "Resilience Engineering"[Title/Abstract] OR "Psychological Solvency"[Title/Abstract] OR "Ethical Unburdening"[Title/Abstract]))

**2. Scopus (via Elsevier)**

- **Search Syntax: n = 362**

(TITLE-ABS-KEY (“Intensive Care" OR "Critical Care" OR "ICU”) AND TITLE-ABS-KEY (“Family Presence" OR "Family Engagement" OR "Surrogates" OR "Caregivers”) AND TITLE-ABS-KEY ("Family Burnout" OR "Sleep Deprivation" OR "Vigilance" OR "Caregiver Burden" OR "PICS-F" OR "Compulsive Hyper-engagement" OR "Clinical Titration" OR "Resilience Engineering" OR "Psychological Solvency”)) AND PUBYEAR < 2027

**3. Web of Science Core Collection (via Clarivate Analytics)**

- **Search Syntax: n = 797**

TS= (("Intensive Care" OR "Critical Care" OR "ICU") AND ("Family Presence" OR "Family Visitation" OR "Surrogate*" OR "Caregiver*") AND ("Vigilance" OR "Caregiver Burden" OR "Sleep" OR "Anxiety" OR "PICS-F" OR "Compulsive Hyper-engagement" OR "Clinical Titration" OR "Resilience" OR "Allostatic Overload"))

**Search Terms Refinement: Demarcating Established vs. Proposed Constructs**

| **Concept Domain** | **Established Keywords & MeSH Terms** | **Proposed Theoretical Constructs (Innovation)** |
| --- | --- | --- |
| **Setting (ICU)** | Intensive Care Units [MeSH], Critical Care, ICU. | Green ICU, Sustainable Care Ecosystem. |
| **Population** | Family [MeSH], Caregivers [MeSH], Surrogates, Relatives. | Capacitated Partners, Family Unit. |
| **Phenomenon** | Vigilance, Caregiver Burden, Sleep Deprivation, Unregulated Visitation. | **Compulsive Hyper-engagement**, **Clinical Titration of Presence**. |
| **Outcomes** | PICS-F, Anxiety, PTSD, Allostatic Overload. | **Active Resilience Engineering**, Psychological Solvency, **Ethical Unburdening**. |

**Methodological Transparency (JBI Three-Step Approach)**

1. **Phase I: Initial Exploration:** A preliminary search was conducted on PubMed and Google Scholar to identify index terms and keywords within relevant titles and abstracts, ensuring the search captured the "Pathophysiology of Depletion" phenomenon.
2. **Phase II: Systematic Identification:** A comprehensive search using the identified keywords and index terms was conducted across the specified platforms (NLM, Elsevier, and Clarivate) to retrieve evidence published up to February 22, 2026.
3. **Phase III: Reference Chaining:** To mitigate "Search Strategy Bias," the reference lists of all included studies were hand-searched (Snowballing) to identify supplementary literature that utilizes alternative terminology for the surrogate resilience process.

To maintain data integrity during the export process, Python scripts (utilizing the Pandas library and respective Application Programming Interface (API) wrappers) were utilized to extract, aggregate, and format the metadata into comma-separated values (CSV) files.
